# Significant Lead Migration of a Subcutaneous Implantable Cardioverter-Defibrillator in a Pediatric Patient

**DOI:** 10.19102/icrm.2017.080101

**Published:** 2017-01-15

**Authors:** Nayan T. Srivastava, Adam C. Kean

**Affiliations:** ^1^Department of Pediatric Cardiology, Indiana University School of Medicine, Indianapolis, IN

**Keywords:** Lead migration, pediatric, subcutaneous intracardiac defibrillator

## Abstract

Since its introduction, the subcutaneous implantable cardioverter-defibrillator (S-ICD) has provided the benefit of reduced mortality from ventricular tachyarrythmias without the associated short- and long-term morbidity of transvenous or epicardial implantable cardioverter-defibrillator (ICD) leads. As its name implies, the S-ICD system is implanted in its entirety, including device and lead, just under the skin beginning along the anterior axillary line, with its lead tunneled to the left parasternum and then from the xiphoid to the manubrium–sternal junction. Dislocation of the lead due to migration of the parasternal lead has been described in a minority of patients. Here, we describe an unusual case of a significant lead migration in a pediatric patient.

## Case presentation

The patient was a 16-year-old male with hypertrophic cardiomyopathy (HCM) who initially presented to his primary cardiologist after two episodes of syncope while playing competitive basketball. He had a muscular build including weight 86.9 kg (92nd percentile), stature 185.5 cm (92nd percentile), and body mass index 29 (96th percentile). His baseline electrocardiogram showed a sinus bradycardia at 51 bpm with massive left-ventricular hypertrophy and T-wave inversion in the lateral precordial leads V4–V6 as well as II, III, and aVF. An echocardiogram showed asymmetric hypertrophy of the posterior free wall and apex and normal diastolic intraventricular wall thickness of 15 mm. He was initiated on metoprolol XL and restricted from activities. Further evaluation included a Holter monitor, exercise treadmill test and cardiac magnetic resonance imaging, which demonstrated non-specific atrial and ventricular ectopy, normal blood pressure response, and no myocardial late gadolinium enhancement, respectively. Genetic testing was similarly negative for Class I and Class II mutations, although a Class III mutation was present in the GLA gene. Despite the mixed diagnostic studies, in the presence of unexplained syncope and adequate clinical criteria for HCM, he was referred for placement of a primary prevention ICD.

Upon referral to our office, we reviewed the clinical indication for device placement and described the treatment options including a transvenous single-chamber single-coil ICD system and S-ICD system. Given the patient and family’s interest in the S-ICD system, he underwent electrode screening supine, sitting, standing and while using a stationary bicycle. He was determined to be an appropriate candidate for the S-ICD system.

**Figure 1:**
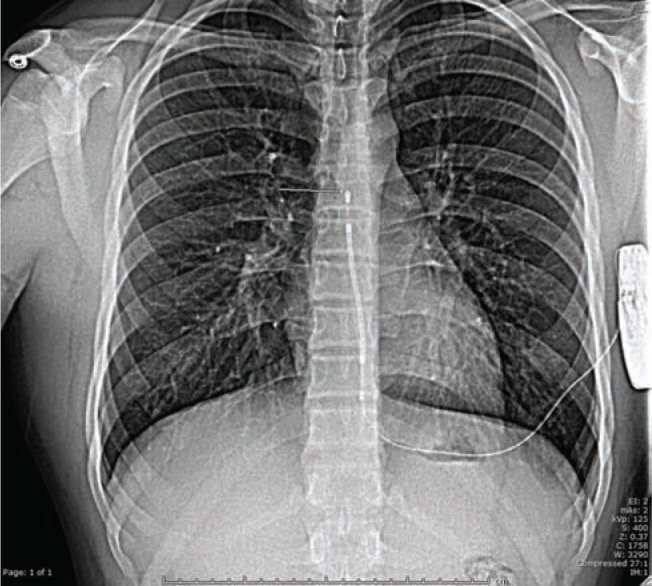
Note the proper post-surgical positioning of the lead.

The procedure was performed under general anesthesia in the pediatric electrophysiology laboratory. Given the patient’s large and muscular build, we elected to perform a three-incision technique rather than a two-incision to provide additional lead support. Therefore, a 6-cm incision was made just inferior to the subpectoral groove to the anterior axillary line. A pocket was formed from the incision cranially and laterally, reaching the leftmid-axillary line. A vertical 3-cm incision was made at the left parasternal border at the level of the xiphoid process, exposing the fascia overlying the sternal periostium. The tunneling tool was used to connect this incision to the generator pocket. The lead was then connected to the tunneling tool with a silk tie and pulled from the generator pocket to the xiphoid pocket. The lead was secured to the chest wall fascia with a suture sleeve and a horizontal 3-cm incision was then made at the left upper sternal border to expose the fascia overlying the sternal periostium. The tunneling tool was then used to connect the inferior pocket to the lead tip pocket and the lead was pulled through to the superior position. The lead pockets were sutured closed, and the lead was connected to the generator. Sensing vectors were tested and the configuration was set at the alternate vector. Defibrillation testing was successful on the first treatment of 65 J. The device was programmed with a conditional shock zone 220 bpm and shock zone 240 bpm, and skin closures were then completed. The patient was moved to the postanesthesia care unit in stable condition. A post-surgical anterior–posterior (AP) projection chest X-ray was completed, which showed stable positioning of the device and lead. The following morning, a repeat (posterior–anterior) PA chest X-ray showed the tip of the lead at roughly the seventh rib consistent with the fluoroscopic positioning from the prior day **(Figure 1)**.

The patient was evaluated in the emergency department (ED) for a non-specific fever approximately 10 days after the procedure. The work-up was unremarkable, with eventual resolution of the fever and a non-concerning surgical site. An X-ray obtained during this visit showed stable location of the device and leads. The patient was discharged with a routine follow-up schedule ordered.

**Figure 2:**
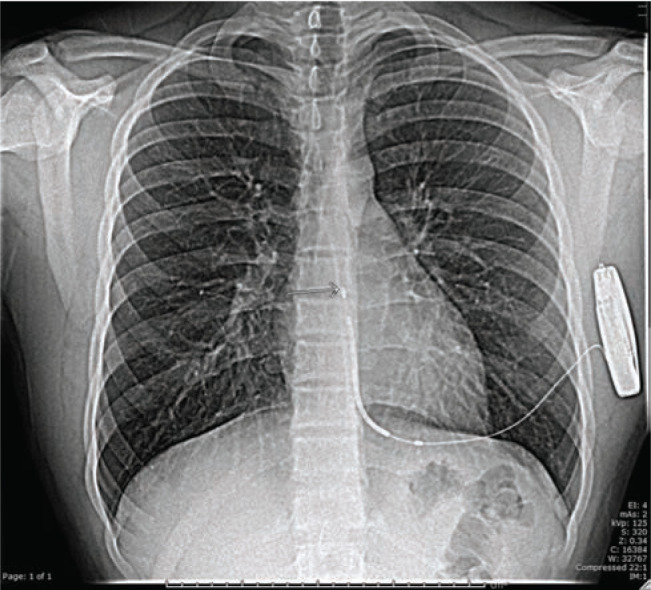
Chest X-ray showing lead migration with displacement of approximately 4.5 cm.

The patient was seen six weeks later for routine follow-up, where he reported an ongoing feeling of contact between the device and his left scapula upon upward stretching of his left arm, as well as a specific episode of stretching during which he felt a tug from his sternum to the left axilla and then relief of the pulling sensation. This episode had occurred some time after he was evaluated in the ED for fever. A chest X-ray performed at this time showed that the lead had migrated from a purely vertical position to a hockey stick right contour with a displacement of approximately 4.5 cm with the tip overlying rib 9 **(Figure 2)**. Evaluation of the patient’s device demonstrated adequate sensing, though with a change in the optimal vector from alternate to secondary. The sensing configuration was changed to the secondary vector and the decision was made to obtain a chest X-ray in four weeks for evidence of any continued lead migration.

**Figure 3:**
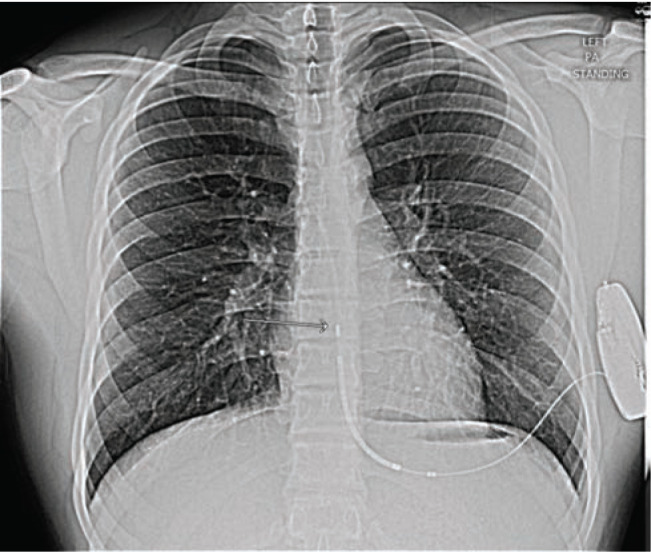
Further regression of the lead to a more L-shaped configuration.

Owing to social developments, the chest radiograph was not completed at that time. Follow-up after an additional 2 months showed further regression of the lead with the lead tip sitting at approximately 6.5 cm below its original position with an increased L-shaped configuration of the high voltage coil **(Figure 3)**. Again, vector analysis showed adequate sensing with no change in lead impedance. Despite a continued lack of symptoms and arrhythmias, based on the continued movement of the lead, there was a growing question of progressive failure to sense. Moreover, no data regarding defibrillation success was available with the new position. As such, the decision was made to reposition the lead.

The patient was taken back into the operating room for lead revision approximately 5 months following the initial system placement. We incised the inferior and superior sternal scars and dissected down to the fascia overlying the periosteum. The tip of the lead was not present at the upper incision, nor was the suture sleeve present at the lower. Retained non-absorbable suture material tied to the fascia was present at both sites.

The tip of the lead was withdrawn to the lower incision and fully extended. The suture sleeve was not visible and presumably retained proximal to the lower incision site.

At this time, the tip of the lead was secured to a new tunneling tool and extended to the superior incision. Its placement was verified with fluoroscopy and the lead tip was then secured at the superior incision site with two non-absorbable stay sutures and the proximal electrode secured with a new suture sleeve and two non-absorbable stay sutures as well. The pockets were then closed in the usual fashion. The sensing vector of choice returned to the alternative vector. Defibrillation testing was not repeated given the prior success of leads and device in the same positions as confirmed by radiography. The patient tolerated the procedure without difficulty and recovered without any fever, pain, swelling, or induration. He limited his activities overall per standard HCM precautions as well as with specific emphasis to avoid activities of the upper extremity to avoid further dislodgement of the lead until adequate healing had taken place.

The patient returned for a routine follow-up visit approximately five weeks after the lead revision. An examination showed healing incisions at the manubrium and xiphoid process. The lateral incision was also wellhealed and the subcutaneous ICD lead and device were palpated and in place. Interrogation of the device showed no treated or untreated episodes of ventricular tachycardia. A chest X-ray showed stable placement of both the lead and generator.

## Discussion

Children and young adults with a history of cardiac arrest, refractory ventricular tachycardia or high risk of sudden cardiac death may benefit from the implantation of an ICD, and children overall have appropriate rates of discharge comparable to the adult population.^1^ However, ICD placement in the pediatric patient may be more challenging due to expected life span requiring long-term device integrity, expected growth and activity level, and/or coexisting congenital heart disease or cardiomyopathy. Transvenous or epicardial ICD in particular carries a risk of long-term intravascular lead complications, including infection, insulation damage and conductor coil breakage.^2^ Other perioperative and postoperative complications such as lead dislodgement, pneumothorax, cardiac perforation and pericardial effusions have also been described.^2^ Moreover, ICD implantation in the pediatric population may be limited by patient size, venous anatomy and/or cardiac anatomy.^3^ In children of adult size, a two-incision technique may be employed to implant the S-ICD, thereby avoiding the superior parasternal incision. However, a three-incision technique was employed for this particular patient given his large and muscular build, in hopes of providing additional lead support during the scarring process. Despite this modification, however, the lead position moved.

S-ICD systems were developed to minimize the risks associated with transvenous and epicardial ICD placement. Because of their subcutaneous extrathoracic location, there is no lead placed in or on the heart. Therefore, S-ICD devices cannot provide antitachycardia pacing, advanced diagnostic or interrogation capabilities.^2^ Adverse events of S-ICD include pocket infection, skin erosion and need for lead revision.

Lead migration has been described in a minority of patients who have undergone S-ICD placement **(Tables 1 & 2)**.^2,4–12^ When reported, these lead migrations appear to be within 1 cm to 2 cm of the original location, or along the parasternal border. There are very limited reports of pediatric S-ICD placement and, to our knowledge, there are no described cases of significant lead migration in a pediatric patient.

Options for the management of lead migration include observation at regular intervals, more frequent follow-up with device testing and/or repositioning of the subcutaneous lead back to their original location. It is unclear if repositioning is necessary if vector analysis and impedance are adequate.

In this case, the gradual movement of the lead to a horizontal plane 6.5 cm away from its original location raised concern of additional movement. Although device testing indicated adequate vectors, it was unclear if at some point the vectors would become inadequate for proper sensing and defibrillation. The adolescent’s parents were also appropriately concerned about the location of the lead, and provided input into the management decision.

Patients are advised to limit arm movement for approximately 7 to 10 days following placement of an S-ICD. Adherence to this recommendation could be difficult for the pediatric population overall, and particularly in the case of younger children. In this instance, both the end and stay sutures failed secondary to considerable lead tension during patient stretching. Therefore, clinicians, particularly pediatric cardiologists, should be familiar with this possible adverse event.

## Figures and Tables

**Table 1:** Summary of Reported Lead Migrations as a Complication of S-ICD Placement in Adult Patients

Study	Patients Implanted	Patients with Lead Migration	Outcome of Lead Migration	Intervention
Abkenari, et al.^4^	31	2 (6.5%)	Inappropriate shocks in one patient; incidental finding in one patient.	Repositioning of wires with addition of dedicated suture sleeve.
Aydin, et al.^5^	40	0 (0%)		
Bardy, et al.^6^	52 (in European trials).	6 (11.5%); 2 (minor migration); 3 (parasternal dislodgement due to inadequate anchoring of distal electrode); 1 (lead dislodgement at six months during physical activity).		No intervention listed (for minor); lead reposition within one week for three patients with distal lead dislodgement.
Kobe, et al.^7^	69	0 (0%)		
lambiase, et al.^8^	456	4 (<1%)		Two required re-position; two required no action.
Olde Nordcamp, et al.^9^	177	3 (1 cm to 2 cm migration) (1.7%).	Inappropriate sensing; inappropriate shocks in two patients.	Repositioning of wires with addition of suture sleeve.
Weiss, et al.^2^	321	1%*, electrode or generator movement		

*The authors reported that migration did not occur in 99% of patients.

**Table 2:** Summary of Reported Lead Migrations as a Complication of S-ICD Placement in Pediatric Patients

Study	Patients Implanted	Patients with Lead Migration	Outcome of Lead Migration	Intervention
Gradaus, et al.^10^ Jarman, et al.^11^ McLeod, et al.^12^	2 (12 and 14 years old). 16 (10 to 48 years old). 2 (10 and 12 years old).	0 (0%) 0 (0%) 0 (0%)	- - -	- - -
